# PITPNC1 Suppress CD8^+^ T cell immune function and promote radioresistance in rectal cancer by modulating FASN/CD155

**DOI:** 10.1186/s12967-024-04931-3

**Published:** 2024-01-30

**Authors:** Junxian Liang, Limin Liao, Lang Xie, WenWen Tang, Xiang Yu, Yinghao Lu, Hongzhen Chen, Juanli Xu, Lei Sun, Huanmei Wu, Chunhui Cui, Yujing Tan

**Affiliations:** 1 Department of General Surgery, Zhujiang Hospital, Southern Medical University, Guangzhou, China; 2 Department of Radiation Oncology, Zhujiang Hospital, Southern Medical University, Guangzhou, China; 3 Department of General Surgery & Guangdong Provincial Key Laboratory of Precision Medicine for Gastrointestinal Tumor, Nanfang Hospital, Southern Medical University, Guangzhou, China

**Keywords:** PITPNC1, CD8^+^ T cell, Immunosuppression, Rectal cancer, Radioresistance

## Abstract

**Background:**

Radioresistance is a primary factor contributing to the failure of rectal cancer treatment. Immune suppression plays a significant role in the development of radioresistance. We have investigated the potential role of phosphatidylinositol transfer protein cytoplasmic 1 (PITPNC1) in regulating immune suppression associated with radioresistance.

**Methods:**

To elucidate the mechanisms by which PITPNC1 influences radioresistance, we established HT29, SW480, and MC38 radioresistant cell lines. The relationship between radioresistance and changes in the proportion of immune cells was verified through subcutaneous tumor models and flow cytometry. Changes in the expression levels of PITPNC1, FASN, and CD155 were determined using immunohistochemistry and western blotting techniques. The interplay between these proteins was investigated using immunofluorescence co-localization and immunoprecipitation assays. Additionally, siRNA and lentivirus-mediated gene knockdown or overexpression, as well as co-culture of tumor cells with PBMCs or CD8^+^ T cells and establishment of stable transgenic cell lines in vivo, were employed to validate the impact of the PITPNC1/FASN/CD155 pathway on CD8^+^ T cell immune function.

**Results:**

Under irradiation, the apoptosis rate and expression of apoptosis-related proteins in radioresistant colorectal cancer cell lines were significantly decreased, while the cell proliferation rate increased. In radioresistant tumor-bearing mice, the proportion of CD8^+^ T cells and IFN-γ production within immune cells decreased. Immunohistochemical analysis of human and animal tissue specimens resistant to radiotherapy showed a significant increase in the expression levels of PITPNC1, FASN, and CD155. Gene knockdown and rescue experiments demonstrated that PITPNC1 can regulate the expression of CD155 on the surface of tumor cells through FASN. In addition, co-culture experiments and in vivo tumor-bearing experiments have shown that silencing PITPNC1 can inhibit FASN/CD155, enhance CD8^+^ T cell immune function, promote colorectal cancer cell death, and ultimately reduce radioresistance in tumor-bearing models.

**Conclusions:**

PITPNC1 regulates the expression of CD155 through FASN, inhibits CD8^+^ T cell immune function, and promotes radioresistance in rectal cancer.

**Supplementary Information:**

The online version contains supplementary material available at 10.1186/s12967-024-04931-3.

## Introduction

Colorectal cancer (CRC) ranks third in prevalence among all malignant tumors. Despite the widespread application of conventional chemotherapy and radiotherapy for the treatment of CRC, the therapeutic outcomes remain unsatisfactory, leading to high mortality and low 5-year overall survival rates [1]. Radiotherapy is important for improving local control and resection rates, and alleviating local symptoms in the treatment of rectal cancer (RC). Radioresistance not only affected the efficacy of treatment but also increased the risk of surgical complications and radiation side effects. It might also delay the timing of surgery, leading to tumor progression and life-threatening situations [2–4]. Although radiation combined with immunotherapy benefited some patients with radioresistance, for the majority of patients, it not only failed to achieve therapeutic effects, but also exposed patients to the toxic side effects of immunotherapy [5, 6]. Previous research suggested that the molecular mechanisms involved in radioresistance in RC included enhanced DNA damage repair capacity, resistance to oxidative stress, metabolic reprogramming, and cell cycle arrest [7, 8]. However, the role of immune regulatory mechanisms in radioresistance and the predictive biomarkers for the efficacy of combined radiotherapy and immunotherapy remains unclear.

A recent study suggested that radiation therapy could enhance tumor immunogenicity by upregulating tumor cell major histocompatibility complex class I (MHC-I), inducing immunogenic cell death, promoting the maturation of dendritic cells (DCs), recruiting tumor antigen-specific T cells, and activating the type I interferon-mediated signaling pathway. This ultimately enhanced anti-tumor immunity [9]. Although radiotherapy could improve the tumor immune microenvironment and promote tumor cell apoptosis, after the development of radioresistance, an inhibitory immune microenvironment might enhance tumor growth. Radioresistance not only promoted the recruitment of suppressive bone marrow and T regulatory cells but also caused excessive secretion of IFN-γ, which upregulated immune inhibitory ligands like PD-L1 (programmed cell death ligand 1), thereby inhibiting effector T cell function [10]. Immune inhibitory receptors including cytotoxic T lymphocyte antigen 4 (CTLA-4), PD-1, T cell immunoglobulin, and ITIM Domain (TIGIT) and lymphocyte activation gene 3 (LAG-3) constrain T cell function [11–13]. The poliovirus receptor (PVR or CD155), a member of the adhesion molecule family, serves as a ligand for TIGIT and is highly expressed on the surface of various tumor cells, inhibiting the activation of T and NK cells, thus promoting tumor metastasis and progression [14, 15]. However, the involvement of CD155 in radioresistance in RC remains unclear.

Furthermore, our preliminary research suggested that phosphatidylinositol transfer protein cytoplasmic 1 (PITPNC1) plays a role in the reprogramming of fatty acid metabolism and radioresistance [16, 17]. Although research has indicated that tumor cells can evade immune surveillance by altering their own fatty acid metabolism, increasing the secretion of inflammatory factors, upregulating tumor cell surface ligands, and recruiting immune cells through chemokines to evade immune killing [18–21], it remains unclear whether fatty acids are involved in the immune suppression seen in radioresistance. Studies have shown that the activation of the sterol regulatory element-binding protein 1/fatty acid synthase (SREBP1/FASN) signaling pathway can inhibit the apoptosis of tumor cells induced by radiotherapy [22]. Additionally, CD155 and CD96 promoted chemotherapy resistance in cancer stem cells by regulating mitochondrial fatty acid β-oxidation [23]. Fatty acid synthase (FASN), a fatty acid synthesis enzyme, has increasingly become a potential therapeutic target for many tumors, and elevated FASN expression significantly correlates with tumor promotion and radioresistance [24, 25].

Therefore, the focus of this study was to investigate the immune suppression mechanisms of the lipid metabolism-related genes PITPNC1 and FASN in radiotherapy resistance. Our study aimed to explore the mechanisms by which PITPNC1 and FASN promote radiotherapy resistance by regulating immune cell function. This may provide a new strategy for the treatment of radiotherapy-resistant RC.

## Method and materials

### Human specimens

Tumor sections embedded in paraffin and blood samples collected from patients admitted in Zhujiang Hospital of Southern Medical University. The samples used in this study were collected with informed consent, and the study was approved by the relevant ethics committee (2023-KY-033). Based on the imaging results, determine the patient's sensitivity to radiotherapy, with reference to the New Response Evaluation Criteria in Solid Tumours: Revised RECIST guideline (version 1.1). Sensitivity and insensitivity were classified in this experiment based on specimens, using 30% as the threshold. Pre-radiotherapy tissue specimens were collected for the following experiments [26].

### Cell lines

HT29 and SW480 human tumor cell lines, as well as the MC38 mouse tumor cell line, were purchased from Punois (China). The cells were cultured in a complete medium containing 10 U/ml of penicillin, 10 ug/ml of streptomycin (NCM Biotech, China), and 10% fetal bovine serum (Yeasen, China). The cells were cultured in an incubator under 5% CO_2_ at 37 ℃.

### Irradiation

The HT29, SW480, and MC38 cells were cultivated and exposed to a radiation dose of 6 Gy in 2-week cycles, for a total of 20 weeks. The establishment of radioresistant strains was confirmed using plate cloning, cell proliferation assays, and apoptosis analyses. Both non-resistant and resistant cell strains were irradiated at a dose of 6 Gy. Using the Elekta Synergy linear accelerator, we employed 6 MV X-ray irradiation with a source-to-skin distance of 100 cm and an absorbed dose rate of 600 cGy/min. The culture plate was covered with a compensator made of silicone gel, measuring 1.0 cm in thickness. The radiotherapy-resistant cell strains obtained were named HT29-RR, SW480-RR, and MC38-RR. Similarly, subcutaneous tumor-bearing mice were subjected to radiotherapy at a dose of 6 Gy administered every 3 days, for a total of five cycles.

### Clone formation

After exposing the radioresistant and radiosensitive strains (HT29, SW480, and MC38) to radiation at a dose of 0, 2, 4, 6, 8 Gy, the cells were digested using pancreatic enzymes and plated in a six-well plate at a density of 500 cells per well. Cell medium was replaced every 2–3 days, and the cells were cultured at 37 ℃ with 5% CO_2_ for a duration of 2 weeks. Cells were fixed in 4% paraformaldehyde (Biosharp, China) and stained with crystal violet (Leagene, China). The number of cell clones was determined under a microscope (Leica, Germany).

### Bioinformatic analyses

The microarray data of all cases with radiosensitivity data in GSE56699 was downloaded under the GPL14951 platform (Illumina HumanHT-12 WG-DASL V4.0 R2 expression beadchip) from the Gene Expression Omnibus (GEO, http://www.ncbi.nih.gov/geo). Furthermore, RNA-sequencing expression (level 3) profiles and the corresponding clinical information for RC were downloaded from the The Cancer Genome Atlas (TCGA) dataset (https://portal.gdc.com). Kyoto Encyclopedia of Genes and Genomes (KEGG) Enrichment Analysis was used to screen for immune checkpoint-related genes and analyze the correlation scores between target genes and immune checkpoints. Similarly, the proteins related to fatty acid metabolism were screened. Subsequently, the correlation scores were analyzed between the target genes and fatty acid metabolism pathways. For microarray data, the R package ‘limma’ was used for differential gene analysis. For RNA-seq data, the R package ‘EdgeR’ was used for differential gene analysis. Gene Set Enrichment Analysis (GSEA) was performed using GSEA v4.0.3. Statistical analyses were performed using R software v4.0.3 (R Foundation for Statistical Computing, Vienna, Austria), where a p-value < 0.05 was considered statistically significant. The correlation analysis of different genes was performed by TIMER2.0.

### RNA sequencing

The RNA libraries were sequenced on an Illumina NovaSeq 6000 platform by LC Bio Technology Co., Ltd. (Hangzhou, China). Total RNA was isolated from the samples and purified according to the manufacturer's protocol using TRIzol reagent (Thermo Fisher, USA). The raw sequencing data were filtered to obtain high-quality sequencing data (Clean Data). High-quality sequencing data were mapped to the reference genome of the species using alignment tools. Gene expression quantification, Gene Set Enrichment Analysis (GSEA), differential gene analysis, and enrichment analysis were performed on the data. The accuracy of gene expression levels highly correlated with the completeness of transcript reconstruction. After filtering the low-quality sequences (such as sequencing adapters and low-quality reads) using Cutadapt, the remaining valid data (Clean Data) were obtained. Gene expression analysis primarily focused on the protein-coding genes annotated in the genome (mRNA). The expression levels of genes were quantified, and the correlation between gene expression characteristics within and between groups, as well as differentially expressed genes, was assessed. To quantify gene expression levels, the Fragments Per Kilobase Million (FPKM) value was normalized to the original read count of the gene. The FPKM value represented the expression levels of genes in different samples. The heat map of the clustered differentially expressed genes visually shows their expression patterns in different samples or treatments. To better reflect the clustering expression pattern, log10(FPKM + 1) was used for non-biological replicates and Z-scores (Z_sample-i_ = [(FPKM_sample-i_)-Mean_(FPKM of all samples)_]/[Standard deviation_(FPKM of all samples)_]) were used for biological replicates. Furthermore, boxplots of FPKM values were used to analyze and visualize gene expression levels across different samples, providing an overview of gene expression at the global level. A heatmap was created using the pheatmap package and boxplots were generated using the ggplot package.

### siRNA and plasmid transfection and lentiviral transduction

PITPNC1 and FASN siRNA, and FASN overexpressed plasmids were purchased from GeenPharma Biotech (China). HT29-RR and SW480-RR cells were seeded in a 6-well plate at a density of 2 × 10^5^ cells, and incubated at 37 ℃ for 24 h. Once the cell density reached 70%, the siRNA was mixed with Lipofectamine 2000^™^ (Thermo Fisher Scientific, USA) and DMEM (Gibco, USA) and incubated for 15 min. The mixture was then added to the culture medium.

Lentivirus construction: Prior to transfection, HEK293T cells were seeded at a density of 80–90% in a 10 cm cell culture dish. The lentiviral vector plasmids psPAX2 and pMDG2, along with the target gene plasmids sh-NC, sh-PTPNC1, and sh-FASN (Guangzhou IGE, China) were prepared. Lipofectamine 2000^™^ was used for cell transfection, following the manufacturer’s recommendations. The virus-containing supernatant was collected at 48 and 72 h post-transfection, filtered using a 0.45 μm filter (Beyotime, China), and stored at – 80 ℃. MC38-RR cells were plated in a six-well plate, and upon reaching 50% cell density, lentiviral supernatant and 10 ug/ml polybrene (Solarbio, China) were added for 16–18 h of infection. After 48 h of infection, 2 ug/ml puromycin (Solarbio) was used for selection for 5–7 days. Finally, stabilize cell lines were validated using western blotting and qPCR (Bio-Rad, USA).

### Real-time quantitative PCR

According to the manufacturer's instructions, total RNA was extracted from cultured cells using a TRIzol reagent kit (Takara, Japan) according to the manufacturer’s instructions. Reverse transcription was performed using a cDNA synthesis kit (Vazyme, China). Following this, sample loading was conducted using a qPCR reagent kit (Vazyme, China), and relative fluorescence quantification was performed using a Bio-Rad qPCR instrument. The relative expression levels were normalized to β-actin (GeenPharma, China) using the 2^−ΔΔCt^ method.

### Western blotting

Total protein was extracted from the cells using RIPA lysis buffer (Beyotime, China). The protein concentration was quantified using a dual bicinchoninic acid protein assay kit (Beyotime, China). Protein electrophoresis was performed on 6% and 10% (w/v) sodium dodecyl sulfate–polyacrylamide gels (Vazyme, China). The separated proteins were transferred onto polyvinylidene fluoride membranes (Millipore, USA) and blocked with 5% skim milk powder (Millipore) at room temperature for 2 h. Subsequently, the blocked membranes were incubated with specific primary antibodies overnight at 4 ℃, followed by incubation with secondary antibodies at room temperature for 1.5 h. The following antibodies were used: PITPNC1 (1:800, Sigma, USA), FASN (1:1200, Proteintech, China), CD155 (1:1000, CST, USA), Tubulin (1:5000, Proteintech, China), BCL-2 (1:1000, Abcam, USA), Cleaved Caspase-3 (1:1000, Abcam, USA), Bax (1:1000, Immunoway, USA), and goat anti-rabbit and goat anti-mouse secondary antibody (1:10000, Fude, China). After washing three times for 5 min each with TBST, the proteins were visualized using an ECL chemiluminescent substrate (Millipore, USA) on an Alliance imaging system (UVITEC, UK), and the densities were quantified using ImageJ software. Tubulin served as the internal reference protein.

### Measuring cell proliferation

si-PITPNC1-transfected radioresistant, radioresistant, or radiosensitive tumor cells were seeded at a density of 3000–5000 cells per well in a 96-well tissue culture plate. After 8 h of incubation, irradiation at 6 Gy was administered followed by continued incubation. The proliferation rate was assessed at 72 h, 96 h, and 120 h using a CCK8 assay kit (Dojindo, Japan). Wells containing growth medium without any drugs were used as controls. Luminometric analysis (Biotek, USA) was conducted according to the manufacturer's instructions for CCK8, and proliferation rates were calculated using GraphPad software.

### Co-immunoprecipitation

HT29 or SW480 cells were collected in two 10 cm cell culture dishes and lysis buffer (800 μL) and a protease inhibitor were added. Cells were incubated at 4 ℃ for 30 min followed by centrifugation at 10,000 *g*, 4 ℃ for 15 min to remove insoluble material. Following the manufacturer’s instructions, 1–1.5 mg of total protein was removed and immunoprecipitation (IP) was performed overnight at 4 ℃ using 4 µg of IP antibody of FASN (Proteintech, China). The immune complexes were incubated with 50 µl of Protein G magnetic beads (Proteintech, China) at 4 ℃ for 3 h. The immunoprecipitates were washed three times with buffer and resuspended in 5X sample buffer (Proteintech, China). The samples were boiled for 8 min and analyzed by western blotting.

### Immunohistochemistry and immunofluorescence

For immunohistochemistry, the tissue samples embedded in paraffin were sliced into 4 µm thick sections. The sections were baked at 68 ℃ for 90 min, followed by dewaxing in xylene and rehydration in a gradient of alcohol. Antigen retrieval was performed using a sodium citrate + EDTA buffer (Beyotime, China) at 95–100 ℃ for 8 min, after which the sections were cooled to room temperature. They were then washed three times with PBS for 5 min each, and endogenous peroxidase activity was blocked with 3% hydrogen peroxide (Beyotime, China) for 15 min. Non-specific binding sites were blocked with 5% goat serum (Boster, China) at room temperature for 30 min. Subsequently, the sections were incubated overnight at 4 ℃ with specific primary antibodies: PITPNC1 (1:200, Novus, USA), FASN (1:200, Proteintech, China), CD155 (1:200, Bioss, China), Ki67 (1:200, Thermo Fisher, USA), CD8 (1:200, Abcam, USA), CD11c (1:200, SAB, USA). The following day, the sections were incubated at room temperature with secondary antibodies for 30 min. HRP activity was detected using DAB (Dako, Denmark) and cell nuclei were stained with hematoxylin. Finally, the sections were dehydrated in an alcohol gradient, clarified in xylene, and mounted with a neutral resin. Images were acquired using a Leica DM2500 microscope.

For cellular immunofluorescence or immunofluorescence of tissue sections after antigen retrieval, cells were fixed with 4% paraformaldehyde at room temperature for 20 min, washed, and permeabilized with 0.25% Triton-X-100 (Solarbio, China) at room temperature for 15 min. Subsequently, the slides were blocked with 5% goat serum for 1 h and incubated overnight with the primary antibody. On the following day, the slides were incubated with the fluorescent secondary antibody (1:400, CST, USA) for 1 h, stained with 1 μg/ml DAPI (Solarbio, China) for 15 min, and observed and captured under a Nikon microscope. The antibodies used were PITPNC1 (1:200, Sigma, USA), FASN (1:200, Immunoway, USA), CD155 (1:200, Immunoway, USA), CD8 (1:200, Abcam, USA), IFN γ (1:200, Bioss, China), CD3 (1:200, SAB, USA), CD4 (1:200, Abcam, USA), CD11c (1:200, SAB, USA), CD86 (1:200, Abcam, USA).

### PBMC isolation and co-culture with tumor cells

Informed consent was obtained from all the donors. Peripheral blood mononuclear cells (PBMCs) were obtained by density gradient centrifugation (Solarbio, China) of heparinized venous blood from healthy volunteers. Subsequently, the acquired PBMCs were co-cultured with tumor cells at a ratio of 10:1 for 48 h to assess immune alterations and tumor cell apoptosis.

### CD8^+^ T cell isolation and co-culture with tumor cells

The CD8^+^ T cell fractionation samples were derived from the aforementioned peripheral blood mononuclear cells (PBMC) extraction. Following the operational steps of the sorting reagent kit (STEMCELL, Canada), positive selection was conducted using a magnetic bead-based approach in conjunction with CD8 antibodies. The sorted CD8^+^ T cells were activated for 48 h using CD3/CD28 antibodies (STEMCELL, Canada), and subsequently, cultivation was continued in a serum-free medium (Lonza, Switzerland) supplemented with 20 ng/ml IL-2 (SinoBiological, China). We employed a CD8^+^ T cell to tumor cell ratio of 5:1, and after co-incubation for 48 h, cell collection was performed to analyze the functionality of CD8^+^ T cells and apoptosis of tumor cells. In the analysis of tumor cell apoptosis, a DIR (Beyotime, China) membrane stain was applied to the tumor cells.

### Flow cytometry

To analyze cellular apoptosis after radiotherapy or siRNA transfection, cells were collected by digestion with pancreatic protease (Gibco, USA). After 6 Gy radiotherapy, the cells were collected at 72 h and then washed three times with PBS containing 2% fetal bovine serum. Subsequently, the cells were incubated with Annexin-V-FITC and propidium iodide (PI) (Dojindo, Japan) at 4 ℃ for 30 min. For cell cycle analysis, cells were fixed in 70% cold ethanol for 2 h and stained with PI in the presence of RNase A (Beyotime, China). Fluorescence intensity was measured using FAC scanning (Beckman, USA). Apoptotic cells were defined as annexin V-positive cells. The percentages of cells in the G0-G1, S, and G2-M phases of the cell cycle were counted and compared. Prior to analyzing the immune changes in co-cultured PBMCs, mouse tumor tissues, and peripheral blood, the peripheral blood was treated with red blood cell lysis buffer (Boster, China) at 4 ℃ for 10 min to remove red blood cells. A single-cell suspension of the tumor tissue was prepared by passing it through a 70 µm cell filter. Then, the samples were washed twice with PBS, resuspended in 100 µl of staining buffer, and labeled with monoclonal antibodies specific to various immune cells and cytokines. For cytokine staining, Foxp3/transcription factor staining buffer and intracellular fixation permeabilization buffer (eBioscience, USA) were used. The following flow cytometry antibodies were used in this study: IFN-γ (B27, Biolegend, USA), CD8a (RPA-T8, Biolegend, USA), CD11c (BU15, Elabscience, China), CD45 (HI30, Elabscience, China), CD3 (UCHT1, Elabscience, China), CD86 (GL-1, Elabscience, China), CD86 (BU63, Elabscience, China), CD8a (53–6.7, Elabscience, China), CD4 (GK1.5, Biolegend, USA), CD11c (N418, Biolegend, USA), CD45 (S18009F, Biolegend, USA), and CD3 (17A2, Biolegend, USA).

### In vivo studies

All animal experiments were conducted in accordance with the “Public Health Service Policy on Humane Care and Use of Laboratory Animals” and approved by the Ethics Committee of Zhujiang Hospital, Southern Medical University (LAEC-2022-203). C57BL6/J mice, aged 6–7 weeks, were purchased from the Guangdong Medical Animal Experimental Center. The mice were housed under specific pathogen-free conditions, provided with ad libitum access to food and water, and maintained at a temperature of 22 ℃ with a 12 h light/dark cycle. MC38 stable cell lines were selected for subcutaneous tumor implantation, and the groups were categorized as WT, RR, RR-sh-NC, RR-sh-PITPNC1, RR-sh-FASN, and RR-sh-PITPNC1 + oe-FASN. Additionally, 2 × 10^6^ cells were implanted subcutaneously in each mouse. Radiotherapy was initiated 7 day after subcutaneous tumor implantation. When irradiating the mice, lead plates were used to shield the non-tumor areas.

### Statistical analysis

All data were analyzed using GraphPad Prism software (v 9.0). Results are shown as the mean ± SD from three independent experiments. For comparisons, the t-test, Wilcoxon rank-sum test, chi-squared test, or one-way analysis of variance (ANOVA) was used. Statistical significance was set at p < 0.05.

## Results

### PITPNC1 was associated with resistance to radiotherapy in rectal cancer

**Fig. 1:**
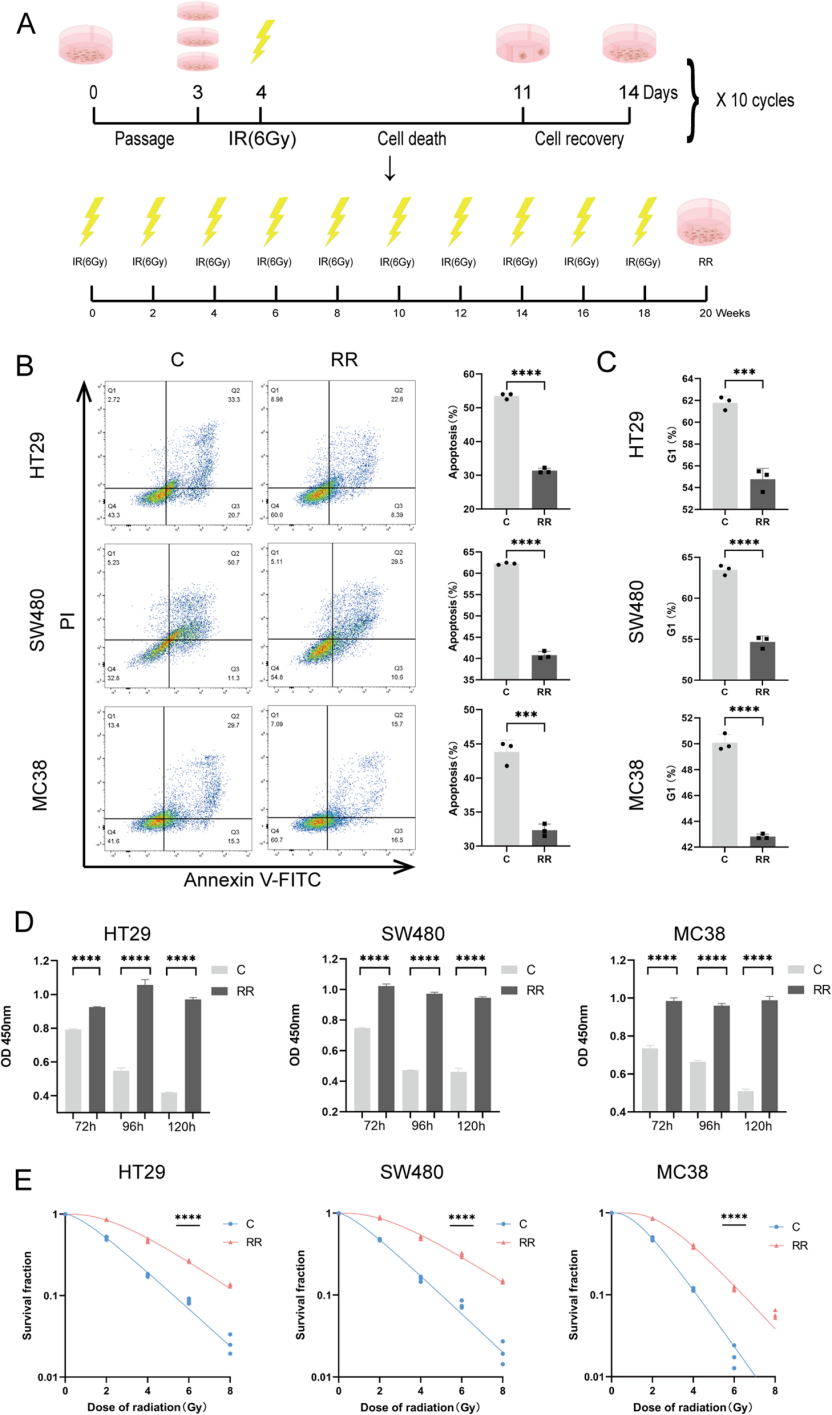
Establishment and validation of radioresistant cell strains. **A** Schematic illustration of radioresistant cell strains construction. **B** Flow cytometry analysis of apoptosis rate in HT29, SW480, and MC38 radioresistant cell strains and radiosensitive groups after 6 Gy radiotherapy. **C** Flow cytometry analysis of cell cycle changes between the two groups after 6 Gy radiotherapy. **D** CCK-8 assay to determine cell inhibition rate of the two groups. **E** Evaluation of colony formation by survival curves. Data indicate the mean ± SD. *p < 0.05, **p < 0.01, and ***p < 0.001, by 2-tailed Student’s t test or one-way ANOVA. N = 3

**Fig. 2:**
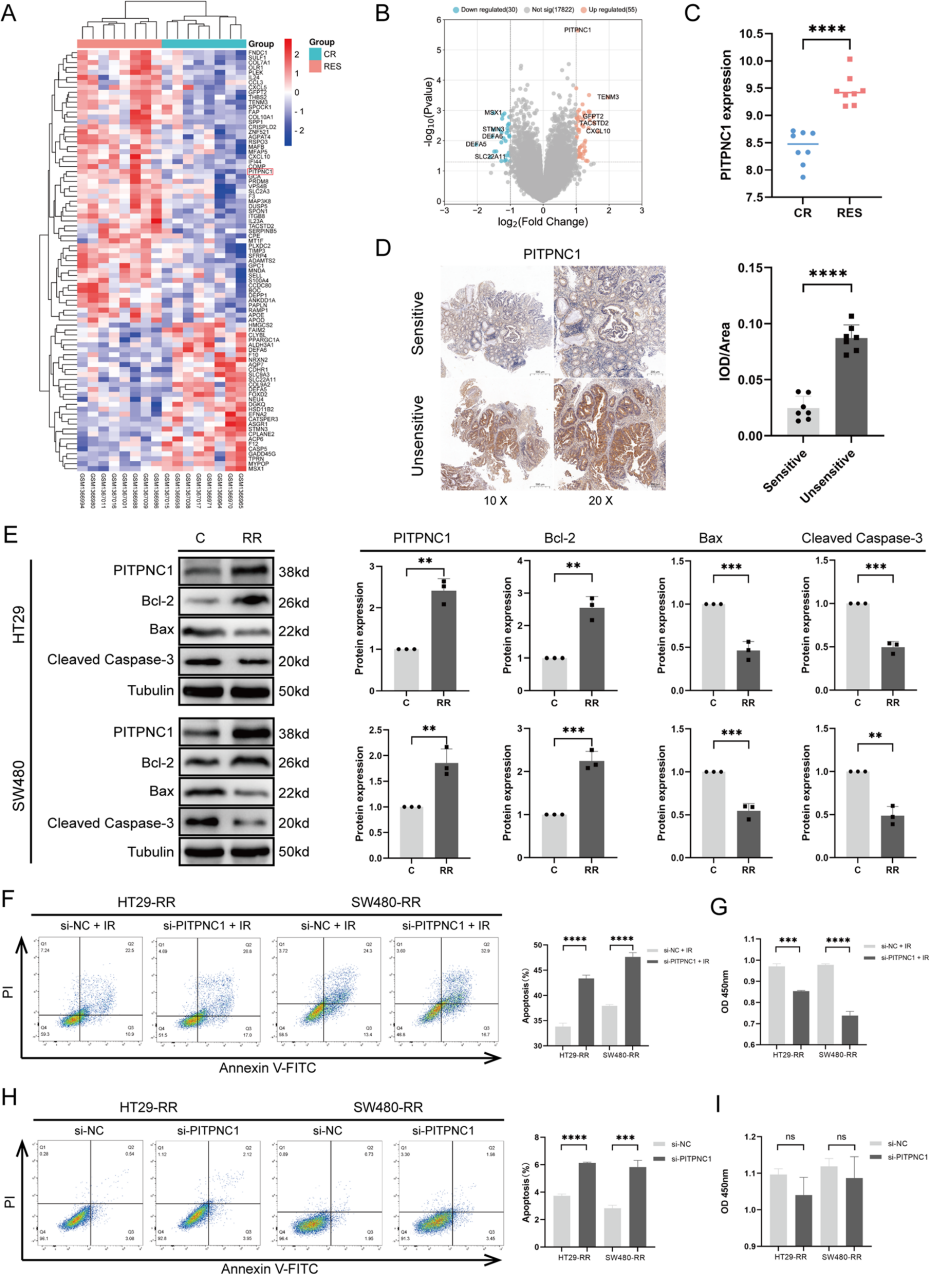
Expression of PITPNC1 and its association with radioresistance in rectal cancer. **A** Differential gene heatmap analysis of expression chip data from 16 patients with rectal cancer. This figure displayed the top 55 up-regulated genes and the top 30 down-regulated genes. The color gradient in the heatmap ranged from blue to red, indicating low to high expression levels, respectively. **B** Volcano plot: The plot was generated using fold change values and p-adjust. Red dots represent upregulated genes, while blue dots represent downregulated genes. **C** Comparing the expression levels of the PITPNC1 in tumor tissues. N = 8. **D** A comparison of PITPNC1 expression levels in sensitive and unsensitive rectal cancer tissues was conducted using immunohistochemistry. N = 7. **E** After 6 Gy irradiation, the expression levels of PITPNC1, Bcl-2, Bax, and Cleaved caspase-3 were detected using western blotting. N = 3. **F** Flow cytometry analysis of cell apoptosis rates treated with 6 Gy irradiation. N = 3. **G** Cell viability assessed by CCK-8 after 6 Gy irradiation. N = 3. **H–I** F and G without 6 Gy irradiation. N = 3. *RES* Resistant, *CR* Complete Remission, *C* Control, *RR* Radioresistance. Data indicate the mean ± SD. *p < 0.05, **p < 0.01, and ***p < 0.001, by 2-tailed Student’s t test or one-way ANOVA

First, we established radioresistant strains using human colorectal cancer cells HT29 and SW480, as well as murine cells MC38. Subsequently, the successful construction of radioresistant strains was validated using cell apoptosis, cell cycle, CCK8 inhibition rate, and colony formation assays (Fig. 1). After irradiation with 6 Gy, compared to the untreated cell line, the radioresistant cell line exhibited a significant decrease in apoptosis rate (Fig. 1B), shortened G1 phase cell cycle arrest (Fig. 1C), and a significantly increased cell proliferation rate (Fig. 1D). The clonogenic curves also demonstrated that the radioresistant cell line was insensitive to irradiation. (Fig. 1E). Previous work by our research team indicated a potential association between the PITPNC1 gene and radioresistance [17]. To further investigate the relevance of PITPNC1 expression levels and radioresistance in rectal cancer, we performed a differential analysis using microarray data from 16 rectal cancer cases (8 radioresistant vs. 8 radiosensitive) from GSE56699. Compared to radiosensitive neoplasms, radioresistant neoplasms exhibited significantly higher expression levels of PITPNC1 (Fig. 2A–C). We examined seven pairs of radiosensitive and unsensitive pre-radiotherapy RC paraffin specimens using immunohistochemical analysis and confirmed high expression levels of PITPNC1 in unsensitive RC (Fig. 2D; Additional file 1: Figure S1A). After irradiation with 6 Gy, western blot experiments revealed increased PITPNC1 expression in the radioresistant strains, whereas the levels of apoptosis-related proteins, Bax and Cleaved Caspase-3, were inversely expressed (Fig. 2E). To further validate the effect of PITPNC1, we used siRNA to knockdown PITPNC1 in radioresistant colorectal cancer cell lines (Additional file 1: Figure S1C). We detected changes in cell apoptosis and proliferation using flow cytometry and CCK8 assay. Knockdown of PITPNC1 in radioresistant strains suppressed cell proliferation and increased the apoptosis rate (Fig. 2F, G). However, when the radiotherapy was withdrawn, we found that the role of PITPNC1 in promoting apoptosis in radioresistant strains was significantly weakened, and its proliferative effect had no statistically significance. (Fig. 2H, I). This suggested that knocking down PITPNC1 could weaken the radioresistance of radioresistant strains, but it did not directly inhibit the proliferation of radioresistant strains significantly.

### Suppression of CD8^+^ T cell immune function enhanced the radioresistance of rectal cancer

**Fig. 3:**
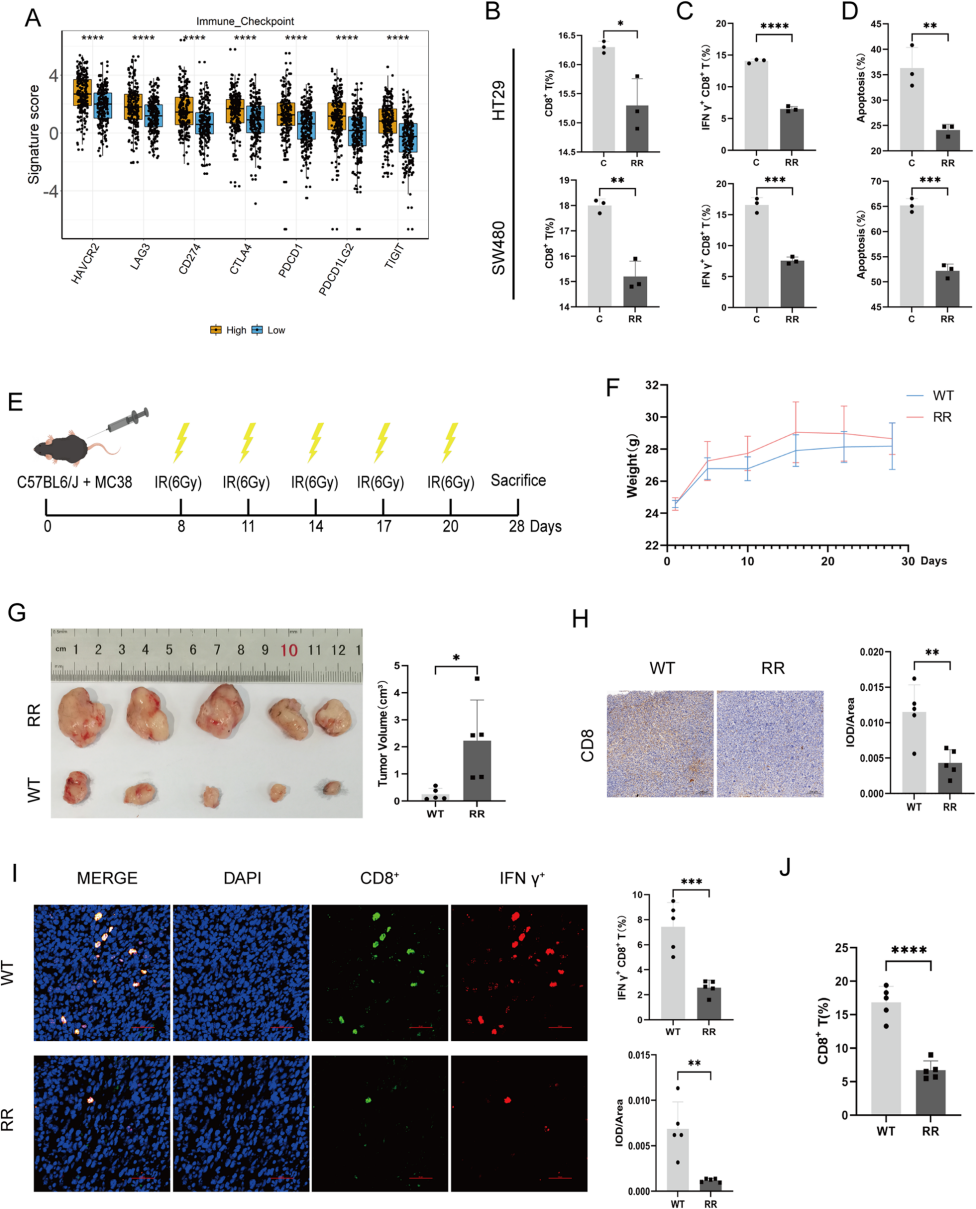
Cells and tumors displaying radioresistance evaded immune eradication by suppressing the immune system. **A** Analysis of the immune-checkpoint-related genes. “High” represented the group with the high expression of PITPNC1, and “low” represented the group with the low. **B** Flow cytometry was performed to analyze the changes in the proportion of CD8^+^ T cells after co-culturing PBMCs with tumor cells. N = 3. **C** The alterations in the proportion of IFN-γ^+^ CD8^+^ T cells assessed by flow cytometry after co-culturing PBMCs with tumor cells. N = 3. **D** The flow cytometry analysis of the apoptosis rate of tumor cells after co-culture. N = 3.**E** Conceptual diagram of the constructed animal model. **F** Graph depicting the weight changes in the animal model. **G** Statistical chart showing the volume of subcutaneous tumors in mice. N = 5. **H** Immunohistochemical analysis of protein expression levels of CD8 in mouse tissues. N = 5. **I** Immunofluorescence analysis of the proportion of IFN-γ^+^ CD8^+^ T cells. N = 5. **J** Flow cytometry analysis of the proportion of CD8^+^ T cells in the tumor tissues. N = 5. *WT* Wild-type group, *RR* Radioresistant group. Data indicate the mean ± SD. *p < 0.05, **p < 0.01, and ***p < 0.001, by 2-tailed Student’s t test or one-way ANOVA

KEGG analysis was performed on rectal cancer data downloaded from the TCGA database. The results indicated a positive correlation between PITPNC1 and the immune checkpoint molecules TIGIT and CTLA-4 (Fig. 3A; Additional file 1: Figure S1B). We co-cultured PBMCs with the HT29-RR and SW480-RR cell lines to validate whether the radioresistant cell lines affected the proportion of various immune cells in PBMCs. Flow cytometry analysis showed that, compared to the radiosensitive group, the proportion of CD8^+^ T cells and IFN-γ^+^ CD8^+^ T cells in the PBMCs of the radioresistant group was significantly decreased, whereas the proportion of DCs showed no significant difference (Fig. 3B, C; Additional file 1: Figure S2A–D). Furthermore, analysis of tumor cell apoptosis in the co-culture system revealed a significant decrease in the apoptosis rate of the radioresistant strains (Fig. 3D, Additional file 1: Figure S2E). In a subcutaneous xenograft model, following five courses of irradiation, we observed a significant increase in tumor volume in radioresistant mice compared to that in wild-type mice (Fig. 3E–G). Immunohistochemical and immunofluorescence staining and flow cytometry analysis of immune cells in the peripheral blood and subcutaneous tumor tissue showed that CD8^+^ T cells and IFN-γ^+^ CD8^+^ T cells were significantly reduced in the tumor tissues of radioresistant mice. However, there were no significant differences in the proportions of immune cells, including peripheral blood immune cells, CD4^+^ T cells and DCs in tumor tissues (Fig. 3H–J; Additional file 1: Figure S3).

### PITPNC1/FASN/CD155 were involved in radioresistance of rectal cancer

**Fig. 4:**
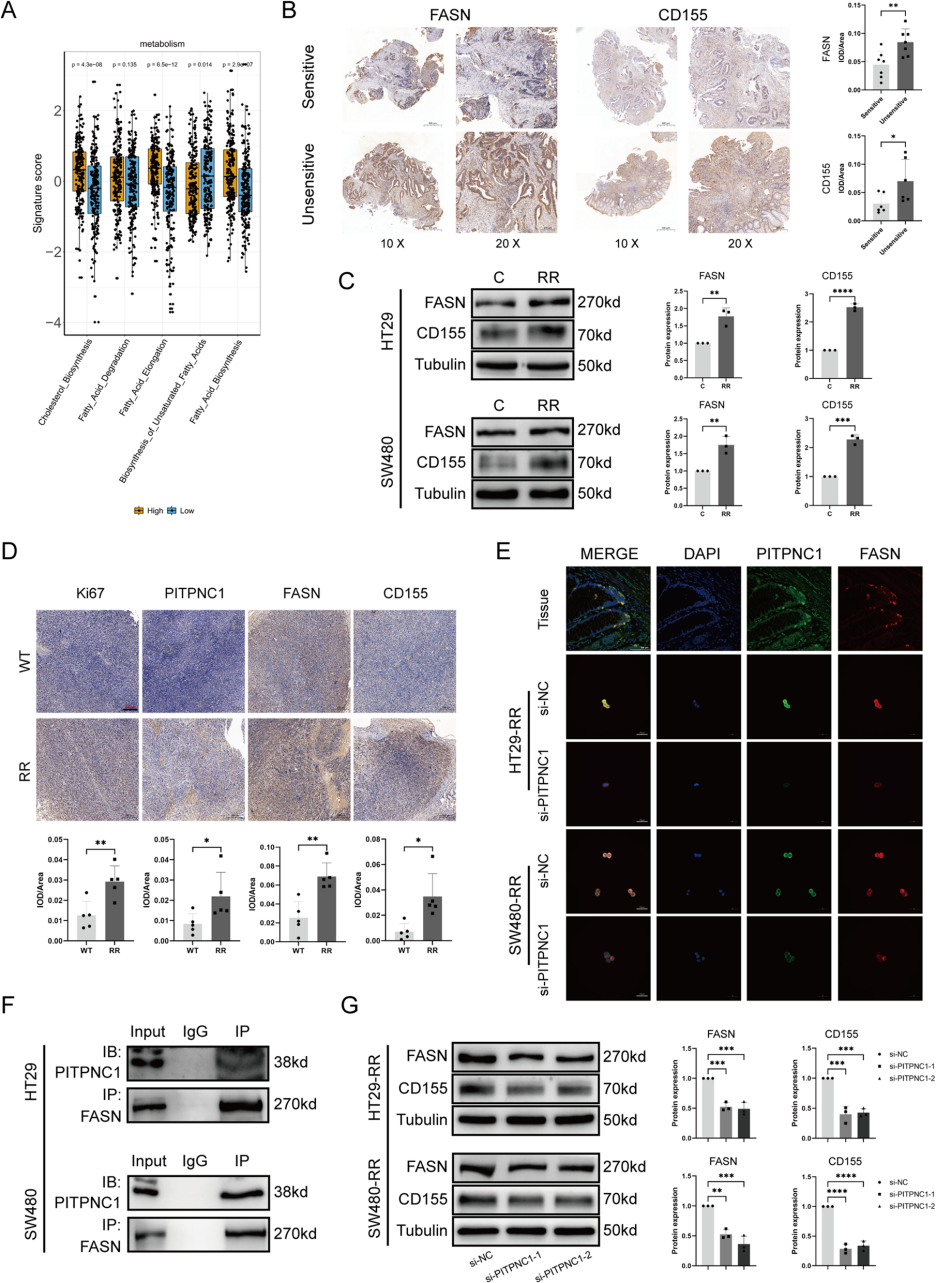
PITPNC1 exhibited a strong positive correlation with the expression of FASN and CD155. **A** Analysis results of fat acid metabolism-associated pathways. “High” represents the group with the high expression of PITPNC1, and “low” represents the group with the low. **B** Immunohistochemical comparison of FASN and CD155 expression levels in radiosensitive and unsensitive rectal cancer tissues. N = 7. **C** Western blotting employed to assess the expression levels of FASN and CD155 in tumor cell lines. N = 3. **D** Immunohistochemical analysis of protein expression levels of Ki67, PITPNC1, FASN, and CD155 in mouse tissues. N = 5. **E** Immunofluorescence co-localization of PITPNC1 and FASN proteins in cells and tissues. **F** CO-IP experiment performed on HT29 and SW480 cell lines to examine the interaction between PITPNC1 and FASN. **G** The expression levels of FASN and CD155 assessed by western blotting after knocking down PITPNC1 in tumor cell lines. N = 3. Data indicate the mean ± SD. *p < 0.05, **p < 0.01, and ***p < 0.001, by 2-tailed Student’s t test or one-way ANOVA

**Fig. 5:**
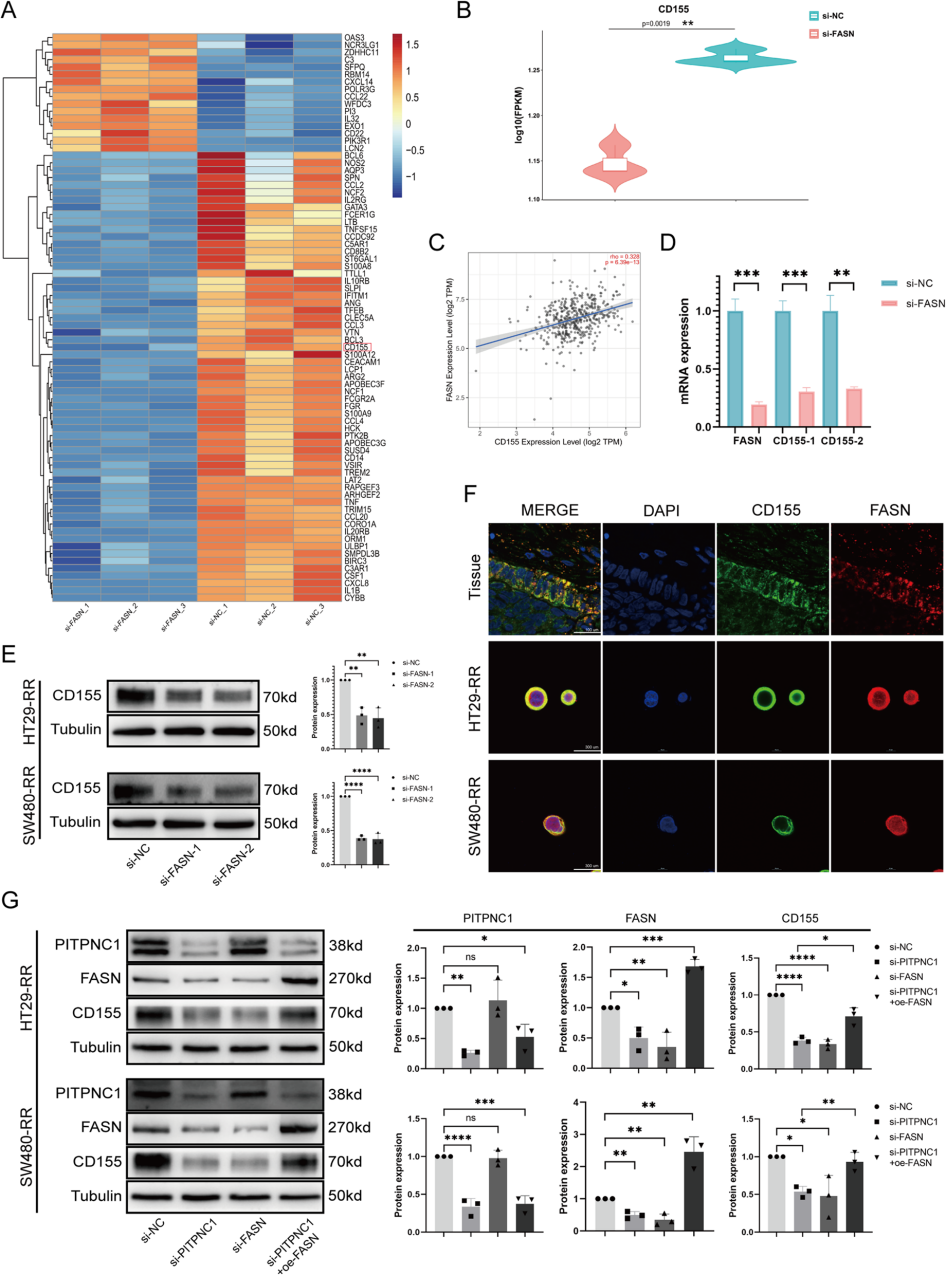
PITPNC1 modulated the upregulation of CD155 via FASN in radioresistant cell lines. **A**, **B** The transcriptome sequencing analysis of SW480-RR cell line after knocking down FASN. The horizontal axis of heatmaps represented the samples and the vertical axis exhibited the genes. The color gradient ranges from blue to red, and this indicated the expression levels from low to high. Box plots were plotted with sample names on the horizontal axis and log10(FPKM) on the vertical axis. **C** TIMER2.0 for the correlation analysis of FASN and CD155 in colorectal cancer. **D** qPCR fluorescence quantification analysis to assess the mRNA expression levels of FASN and CD155 after knocking down FASN. **E** To evaluate the expression levels of CD155 by western blotting after silencing FASN in tumor cell lines. **F** Immunofluorescence co-localization of CD155 and FASN proteins in tissue slides and cells. **G** To assess the expression levels of PITPNC1, FASN, and CD155 in tumor cell lines after individually knocking down PITPNC1 and FASN, as well as overexpressing FASN after PITPNC1 downregulation. Data indicate the mean ± SD. *p < 0.05, **p < 0.01, and ***p < 0.001, by 2-tailed Student’s t test or one-way ANOVA. N = 3

We explored the downstream genes of PITPNC1 by using KEGG analysis of RC data from the TCGA database. These results indicated a positive correlation between the expression of PITPNC1 and fatty acid synthesis (Fig. 4A). Based on a literature review, we identified FASN as a key enzyme involved in fatty acid metabolism [27]. Further analysis of tissue samples by immunohistochemistry confirmed significantly elevated FASN expression in unsensitive RC tissues. Because of the positive correlation between TIGIT and PITPNC1 in the bioinformatics analysis mentioned earlier, we performed immunohistochemical analysis on CD155 (the ligand of TIGIT) in human tissue specimens and found that it was highly expressed in unsensitive tissues (Fig. 4B, C). Immunohistochemical analysis of the two aforementioned groups of mouse tumor tissues showed consistent expression trends of PITPNC1, FASN, and CD155 compared to human tissues (Fig. 4D). To further validate the protein–protein interaction between PITPNC1 and FASN, we performed immunofluorescence co-localization and co-immunoprecipitation, which confirmed a direct interaction between PITPNC1 and FASN (Fig. 4E, F, Additional file 1: Figure S4). After PITPNC1 knockdown, both FASN and CD155 levels consistently decreased (Fig. 4G). To investigate the potential correlation between FASN and CD155, we performed transcriptome sequencing of radioresistant cell lines with FASN knockdown (Additional file 1: Figure S1D). Subsequently, though a heatmap analysis of immune-related genes, the CD155 expression was decreased along with knockdown of FASN (Fig. 5A, B). Next, we utilized the TIMER2.0 database to analyze the correlation between the expression of the FASN and CD155 genes in colorectal cancer tissues, revealing a significant positive correlation (Fig. 5C). qPCR and western blotting were performed to validate the sequencing results (Fig. 5D, E). Additionally, we utilized immunofluorescence to observe a clear co-localization phenomenon between CD155 and FASN, although no direct protein interaction was detected (Fig. 5F). Subsequently, we individually knocked down PITPNC1 and FASN, knocked down PITPNC1 alone, and rescued with overexpressed FASN (Additional file 1: Figure S1E). Western blotting revealed that PITPNC1 regulated the expression of CD155 through FASN (Fig. 5G).

### PITPNC1/FASN/CD155 inhibited the immune function of CD8^+^ T cells

**Fig. 6:**
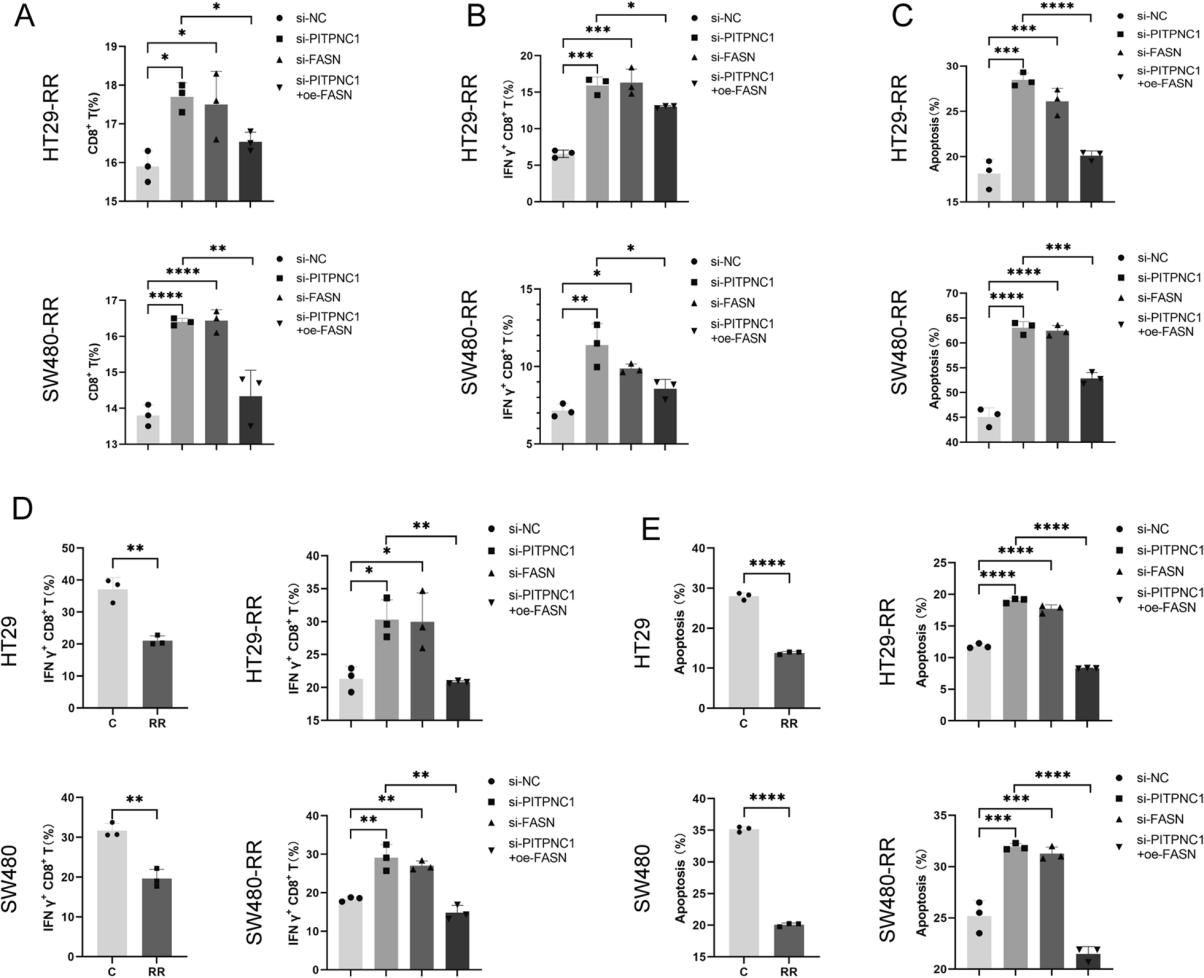
In vitro validation has demonstrated that PITPNC1 governs the upregulation of CD155 via FASN in radioresistant cell lines, leading to immune evasion. **A** The analysis of the proportion of CD8^+^ T cells after co-culturing PBMCs with tumor cells performed by flow cytometry. **B** After co-culturing PBMCs with tumor cells, the flow cytometry analysis demonstrated the changes in the proportion of IFN-γ^+^ CD8^+^ T cells. **C** The flow cytometry analysis of the apoptosis rate of tumor cells in co-cultivation of PBMCs and tumor cells. **D** The alterations in the proportion of IFN-γ^+^ CD8^+^ T cells assessed by flow cytometry after co-culturing CD8^+^ T cells with tumor cells. N = 3. **E** The flow cytometry analysis of the apoptosis rate of tumor cells after co-culture. N = 3. Data indicate the mean ± SD. *p < 0.05, **p < 0.01, and ***p < 0.001, by 2-tailed Student’s t test or one-way ANOVA. N = 3

To investigate the key role of PITPNC1/FASN/CD155 in immune regulation, we individually knocked down PITPNC1 and FASN and knocked down PITPNC1 while overexpressing FASN in radioresistant colorectal cancer cell lines. Subsequently, we co-cultured these tumor cells separately with PBMCs and analyzed their cell profiles in the co-culture system using flow cytometry. The results revealed an increased proportion of CD8^+^ T cells and IFN-γ^+^ CD8^+^ T cells in the co-culture system after the knockdown of PITPNC1 and FASN. However, this immunostimulatory effect was suppressed after the restorative overexpression of FASN (Fig. 6A, B; Additional file 1: Figure S5A, B). Additionally, the analysis of the co-culture system did not reveal any significant differences in the proportion of DCs (Additional file 1: Figure S5C). Simultaneously, examination of the apoptosis rate of tumor cells in the co-culture system indicated an increase in tumor cell apoptosis following the knockdown of PITPNC1 and FASN. Conversely, this enhanced effect on apoptosis was inhibited after the FASN overexpression (Fig. 6C; Additional file 1: Figure S5D).

To assess alterations in PITPNC1/FASN/CD155 within tumor cells and their direct modulation of CD8^+^ T cell functionality, we conducted the isolation of individual CD8^+^ T cells. Upon manipulating the expression of pathway-associated genes in tumor cells and co-cultivating them with CD8^+^ T cells, we observed that downregulating PITPNC1 and FASN led to an increased proportion of IFN-γ^+^ CD8^+^ T cells, along with heightened cytotoxic effects against tumor cells. Conversely, overexpression of FASN resulted in a reversal of this effect (Fig. 6D, E; Additional file 1: Figure S5 E–H).

### PITPNC1 promoted radioresistance in rectal cancer by inhibiting the immune function of CD8^+^ T cells via FASN/CD155 in vivo

**Fig. 7:**
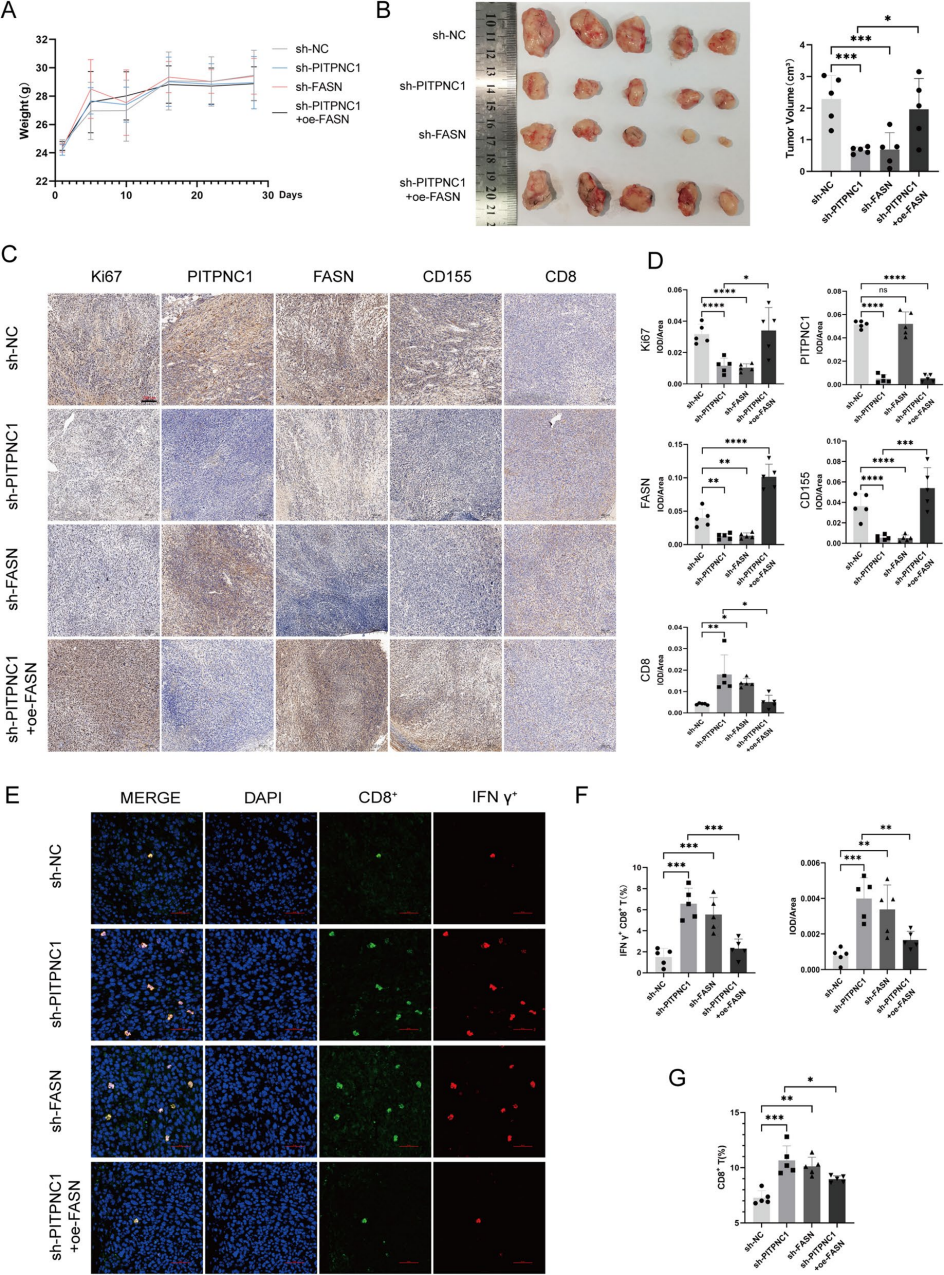
In vivo validation demonstrated that PITPNC1 orchestrates the upregulation of CD155 via FASN in radioresistant tumors, leading to immune evasion. **A** Graph of weight variation in animal models. **B** Statistical chart of subcutaneous tumor volume in mice. **C**, **D** Comparison of protein expression levels of Ki67, PITPNC1, FASN, CD155 and CD8 in mouse tissues through immunohistochemistry. **E**, **F** Immunofluorescence analysis of the proportion of IFN-γ^+^ CD8^+^ T cells in tumor tissues. **G** Analysis of the proportion of CD8^+^ T cells in the tumor tissues using flow cytometry. Data indicate the mean ± SD. *p < 0.05, **p < 0.01, and ***p < 0.001, by 2-tailed Student’s t test or one-way ANOVA. N = 5

After subcutaneous tumor irradiation for five treatment cycles, we observed a significant reduction in tumor volume upon silencing of PITPNC1 and FASN (Additional file 1: Figure S1F). However, this tumor-suppressive effect was weakened after compensatory overexpression of FASN (Fig. 7A, B). Similarly, immunohistochemical analysis revealed that the downregulation of PITPNC1 resulted in decreased expression levels of FASN and CD155, whereas compensatory overexpression of FASN led to an increase in CD155 expression. Immunohistochemical and immunofluorescence and flow cytometry staining demonstrated that the proportion of CD8^+^ T cells and IFN-γ^+^ CD8^+^ T cells in the tumor tissue increased after silencing PITPNC1 and FASN. However, this enhanced immune effect was suppressed after compensatory overexpression of FASN (Fig. 7C–G; Additional file 1: Figure S6A). Moreover, the proportions of immune cells in the peripheral blood and CD4^+^ T cells and DCs in the tumor tissues were not significantly different (Additional files 1: Figures S6B-E and S7).

## Discussion

This study firstly proposed that PITPNC1 promoted radiotherapy resistance in RC by inhibiting the immune function of CD8^+^ T cells through regulation of FASN/CD155. Although our previous studies have reported the association between the PITPNC1 gene and radioresistance, this research delved deeper into this immunosuppressive relationship. The investigation revealed that downregulation of PITPNC1 led to apoptosis in radioresistant cells, whereas direct inhibition of FASN indirectly suppressed the expression of CD155 on tumor cell surfaces. Because CD155 serves as a ligand for CD8^+^ T cell TIGIT, it consequently reduces the immunological recognition of CD8^+^ T cells, indirectly hampering their immune functionality, ultimately resulting in radioresistance. The discovery of PITPNC1 as a key target associated with immunosuppression in radioresistance provided a potential novel target of treatment for patients with radioresistant RC.

In general, previous studies have explored the effects of radiation both in vitro and in vivo, aiming to establish prognostic indicators for radioresistance in patients with cancer and to predict biomarkers. Similarly, in line with the clinical standards for radiotherapy in rectal cancer treatment, we constructed subcutaneous xenografts and executed therapeutic interventions following clinical protocols. Although specific morphological and biochemical criteria have been established to predict the treatment efficacy in clinical practice, the immense challenge of radioresistance remains in the field of radiation oncology. Hence, understanding the molecular mechanisms underlying radioresistance is of paramount significance for formulating clinical strategies for cancer treatment, especially for patients with RC [28–33]. Our findings revealed that, in comparison to the radiosensitive cell strains, the proliferative ability and tumor-forming capacity of radioresistant RC strains substantially increased under radiotherapeutic conditions. However, our understanding of radioresistance and immune escape is limited. Consequently, we embarked on a meticulous exploration of radioresistance and immune evasion.

Currently, owing to radiotherapy resistance, the infiltration of immunosuppressive cells, such as Treg cells and other immune cells, into the tumor microenvironment (TME), as well as the depletion of CD8^+^ T cells, has resulted in the inhibition of anti-tumor immunity [34, 35]. In this study, we performed flow cytometric analysis of tumor tissues from radioresistant and wild-type mice. We observed a decrease in the proportion of CD8^+^ T cells in the immune cell population of radioresistant mice. Concurrently, there was a significant increase in the tumor volume in the radioresistant group. At the same time, some studies suggested that combining radiotherapy with cytokines and/or chemokines, as well as PD-1 and CTLA-4 blockade, could reverse CD8^+^ T cells exhaustion, inhibited myeloid-derived suppressor cells, polarized M2 macrophages, and reduced colony-stimulating factor-1 levels and transforming growth factor β, thereby overcoming tumor radioresistance to some extent [36–41]. Although CD155 functions as an immune checkpoint [14, 42, 43], there have been no reports of its association with radioresistance. Remarkably, our research has shown that reducing the expression of CD155 significantly enhanced IFN-γ production in CD8^+^ T cells and their cytotoxicity against colorectal cancer cells, leading to a marked reduction in radioresistant tumor volume.

Research has indicated that CD47 and CD96 might alter fatty acid oxidation and mediate immune evasion, thereby enhancing tumor radiotherapy resistance [23, 40]. We investigated whether CD155 is also related to fatty acid metabolism, and discovered, that changes in FASN regulated the expression of CD155 on the surface of tumor cells. Recent research suggested that PITPNC1, a lipid transport protein involved in the intermembrane transfer of phospholipids, was of important in modulating β-oxidation of fatty acids in the mitochondria [44–46]. Additionally, our previous work showed that PITPNC1 not only participated in radioresistance but also modulated fatty acid metabolic reprogramming [16, 17]. The current study revealed, for the first time, a direct protein interaction between PITPNC1 and FASN, potentially providing a novel explanation for the role of PITPNC1 in regulating fatty acid metabolism.

## Conclusion

The knockdown of the PITPNC1 gene exhibited the potential to ameliorate radiotherapy resistance. In addition, it enhanced the immune function of CD8^+^ T cells by inhibiting the FASN/CD155. This not only provides a new immunological explanation for PITPNC1-mediated radiotherapy resistance in RC but also establishes a potential theoretical basis for the combination of radiotherapy and immunotherapy.

### Supplementary Information

**Additional file 1: Figure S1.** **A** The radiographic characteristics pre- and post-radiotherapy in sensitive and unsensitive patients, specifically focusing on the tumor shrinkage percentage. N=7. **B **The analysis of the correlation between PITPNC1 and CD8^+^ T cell. Efficiency verification of PITPNC1 (**C**) and FASN (**D**) knocking down or FASN overexpression (**E**) in HT29-RR and SW480-RR cell lines or MC38-RR cell line (**F**). N=3. Data indicate the mean ± SD. *p < 0.05, **p < 0.01, and ***p < 0.001, by 2-tailed Student’s t test or one-way ANOVA. **Figure S2.** **A** Flow cytometry was performed to analyze the changes in the proportion of CD8^+^ T cells after co-culturing PBMCs with tumor cells. **B** The alterations in the proportion of IFN-γ^+^ CD8^+^ T cells assessed by flow cytometry after co-culturing PBMCs with tumor cells. **C**–**D** Analysis of the proportion of DCs in the co-culture through flow cytometry. **E** The flow cytometry analysis of the apoptosis rate of tumor cells after co-culture. Data indicate the mean ± SD. *p < 0.05, **p < 0.01, and ***p < 0.001, by 2-tailed Student’s t test or one-way ANOVA. N=3. **Figure S3.** **A** Flow cytometry analysis of the proportion of CD8^+^ T cells in the tumor tissues. Proportion of CD4^+^ T cells, CD8^+^ T cells (**B**) and DCs **(C**) in the blood of mice analyzed by flow cytometry. Flow cytometry analysis of the proportion of CD4^+^ T cells (**D**) and DCs (**E**) in the tumor tissues from mice. **F** Immunohistochemical analysis of protein expression levels of CD11c in mouse tissues. **G**, **H** Immunofluorescence analysis of CD4^+^ T cells and DCs in mouse tumor tissues. Data indicate the mean ± SD. *p < 0.05, **p < 0.01, and ***p < 0.001, by 2-tailed Student’s t test or one-way ANOVA. N=5. **Figure S4.** Immunofluorescence co-localization of PITPNC1 and FASN proteins in cells. Data indicate the mean ± SD. *p < 0.05, **p < 0.01, and ***p < 0.001, by 2-tailed Student’s t test or one-way ANOVA. N=3. **Figure S5.** **A** The analysis of the proportion of CD8^+^ T cells after co-culturing PBMCs with tumor cells performed by flow cytometry. **B** After co-culturing PBMCs with tumor cells, the flow cytometry analysis demonstrated the changes in the proportion of IFN-γ^+^ CD8^+^ T cells. **C** Analysis of the alterations in the proportion of DCs exhibited by flow cytometry after co-culturing PBMCs with the tumor cells. **D** The flow cytometry analysis of the apoptosis rate of tumor cells in co-cultivation of PBMCs and tumor cells. **E**, **F** After co-culturing CD8^+^ T cells with tumor cells, the flow cytometry analysis demonstrated the changes in the proportion of IFN-γ^+^ CD8^+^ T cells. **G**, **H **The flow cytometry analysis of the apoptosis rate of tumor cells in co-cultivation of CD8^+^ T cells and tumor cells. Data indicate the mean ± SD. *p < 0.05, **p < 0.01, and ***p < 0.001, by 2-tailed Student’s t test or one-way ANOVA. N=3. **Figure S6. A** Analysis of the proportion of CD8^+^ T cells in the tumor tissues using flow cytometry. Analysis of murine blood CD4^+^ T cells, CD8^+^ T cells (**B**) and DCs (**C**) proportions using flow cytometry. **D**, **E** Flow cytometry analysis of CD4^+^ T cells and DCs proportions in murine tumor tissues. Data indicate the mean ± SD. *p < 0.05, **p < 0.01, and ***p < 0.001, by 2-tailed Student’s t test or one-way ANOVA. N=5. **Figure S7. A** Immunohistochemical analysis of protein expression levels of CD11c in mouse tumor tissues. **B**, **C** Immunofluorescence analysis of CD4^+^ T cells and DCs in mouse tumor tissues. Data indicate the mean ± SD. *p < 0.05, **p < 0.01, and ***p < 0.001, by 2-tailed Student’s t test or one-way ANOVA. N=5.

## Data Availability

The original data of RNA sequencing was deposited in GSA database Bioproject and the access number was PRJCA019165. Some of the images were created by Figdraw.

## Abbreviations

PITPNC1: Phosphatidylinositol transfer protein cytoplasmic 1
FASN: Fatty acid synthase
CRC: Colorectal cancer
RC: Rectal cancer
MHC-I: Histocompatibility complex class I
SREBP1: Sterol regulatory element-binding protein 1
PBMCs: Peripheral blood mononuclear cells
TME: Tumor microenvironment
GEO: Gene Expression Omnibus
TCGA: The Cancer Genome Atlas
KEGG: Kyoto Encyclopedia of Genes and Genomes
GSEA: Gene Set Enrichment Analysis
