# Supplementary material for: PITPNC1 Suppress CD8+ T cell immune function and promote radioresistance in rectal cancer by modulating FASN/CD155
Source: J Transl Med. 2024 Jan 30;22:117. doi: 10.1186/s12967-024-04931-3 (PMC10826121; doi:10.1186/s12967-024-04931-3)
Supplement: Supplementary file 1 — Additional file 1: Figure S1. A The radiographic characteristics pre- and post-radiotherapy in sensitive and unsensitive patients, specifically focusing on the tumor shrinkage percentage. N=7. B The analysis of the correlation between PITPNC1 and CD8+ T cell. Efficiency verification of PITPNC1 (C) and FASN (D) knocking down or FASN overexpression (E) in HT29-RR and SW480-RR cell lines or MC38-RR cell line (F). N=3. Data indicate the mean ± SD. *p < 0.05, **p < 0.01, and ***p < 0.001, by 2-tailed Student’s t test or one-way ANOVA. Figure S2. A Flow cytometry was performed to analyze the changes in the proportion of CD8+ T cells after co-culturing PBMCs with tumor cells. B The alterations in the proportion of IFN-γ+ CD8+ T cells assessed by flow cytometry after co-culturing PBMCs with tumor cells. C–D Analysis of the proportion of DCs in the co-culture through flow cytometry. E The flow cytometry analysis of the apoptosis rate of tumor cells after co-culture. Data indicate the mean ± SD. *p < 0.05, **p < 0.01, and ***p < 0.001, by 2-tailed Student’s t test or one-way ANOVA. N=3. Figure S3. A Flow cytometry analysis of the proportion of CD8+ T cells in the tumor tissues. Proportion of CD4+ T cells, CD8+ T cells (B) and DCs (C) in the blood of mice analyzed by flow cytometry. Flow cytometry analysis of the proportion of CD4+ T cells (D) and DCs (E) in the tumor tissues from mice. F Immunohistochemical analysis of protein expression levels of CD11c in mouse tissues. G, H Immunofluorescence analysis of CD4+ T cells and DCs in mouse tumor tissues. Data indicate the mean ± SD. *p < 0.05, **p < 0.01, and ***p < 0.001, by 2-tailed Student’s t test or one-way ANOVA. N=5. Figure S4. Immunofluorescence co-localization of PITPNC1 and FASN proteins in cells. Data indicate the mean ± SD. *p < 0.05, **p < 0.01, and ***p < 0.001, by 2-tailed Student’s t test or one-way ANOVA. N=3. Figure S5. A The analysis of the proportion of CD8+ T cells after co-culturing PBMCs with tumor cells pe [file 12967_2024_4931_MOESM1_ESM.docx]

**Supplementary data**

**
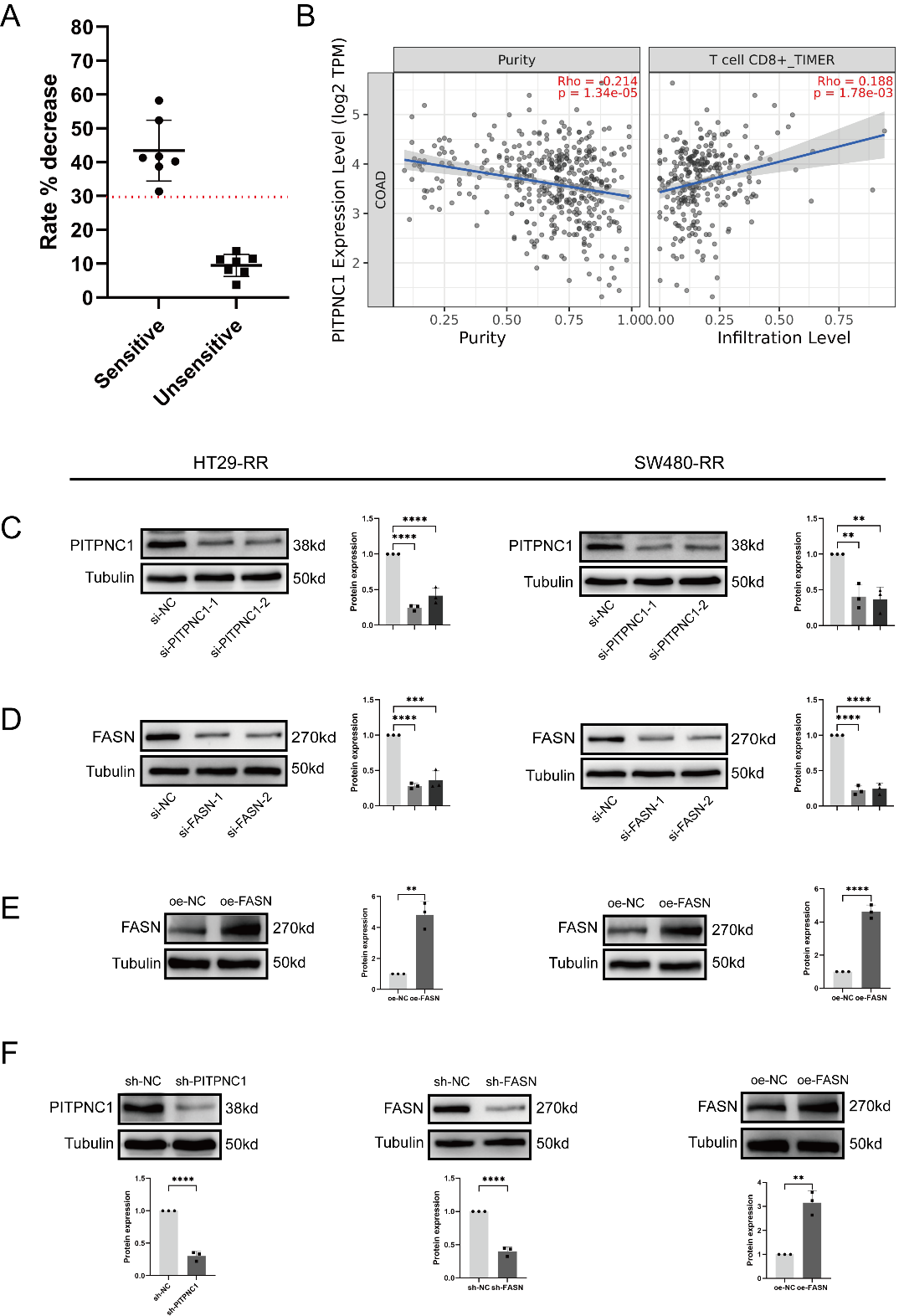
**

**Figure S1.** **(A).** The radiographic characteristics pre- and post-radiotherapy in sensitive and unsensitive patients, specifically focusing on the tumor shrinkage percentage. N=7. **(B).** The analysis of the correlation between PITPNC1 and CD8^+^ T cell. Efficiency verification of PITPNC1 **(C)** and FASN **(D)** knocking down or FASN overexpression **(E)** in HT29-RR and SW480-RR cell lines or MC38-RR cell line **(F)**. N=3. Data indicate the mean ± SD. *p < 0.05, **p < 0.01, and ***p < 0.001, by 2-tailed Student’s t test or one-way ANOVA.


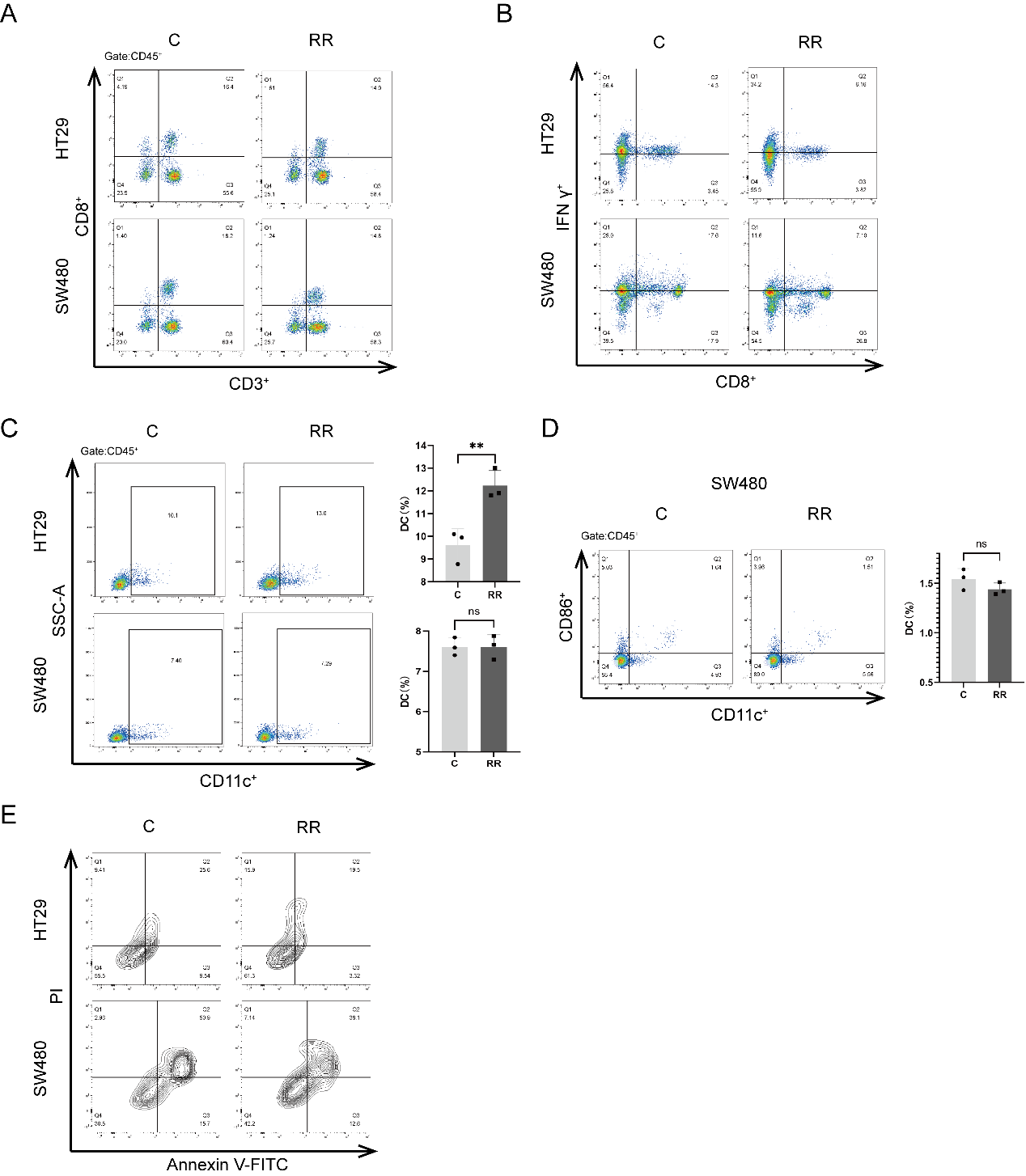


**Figure S2.** **(A).** Flow cytometry was performed to analyze the changes in the proportion of CD8^+^ T cells after co-culturing PBMCs with tumor cells. **(B).** The alterations in the proportion of IFN-γ^+^ CD8^+^ T cells assessed by flow cytometry after co-culturing PBMCs with tumor cells. **(C-D).** Analysis of the proportion of DCs in the co-culture through flow cytometry. **(E).** The flow cytometry analysis of the apoptosis rate of tumor cells after co-culture. Data indicate the mean ± SD. *p < 0.05, **p < 0.01, and ***p < 0.001, by 2-tailed Student’s t test or one-way ANOVA. N=3.


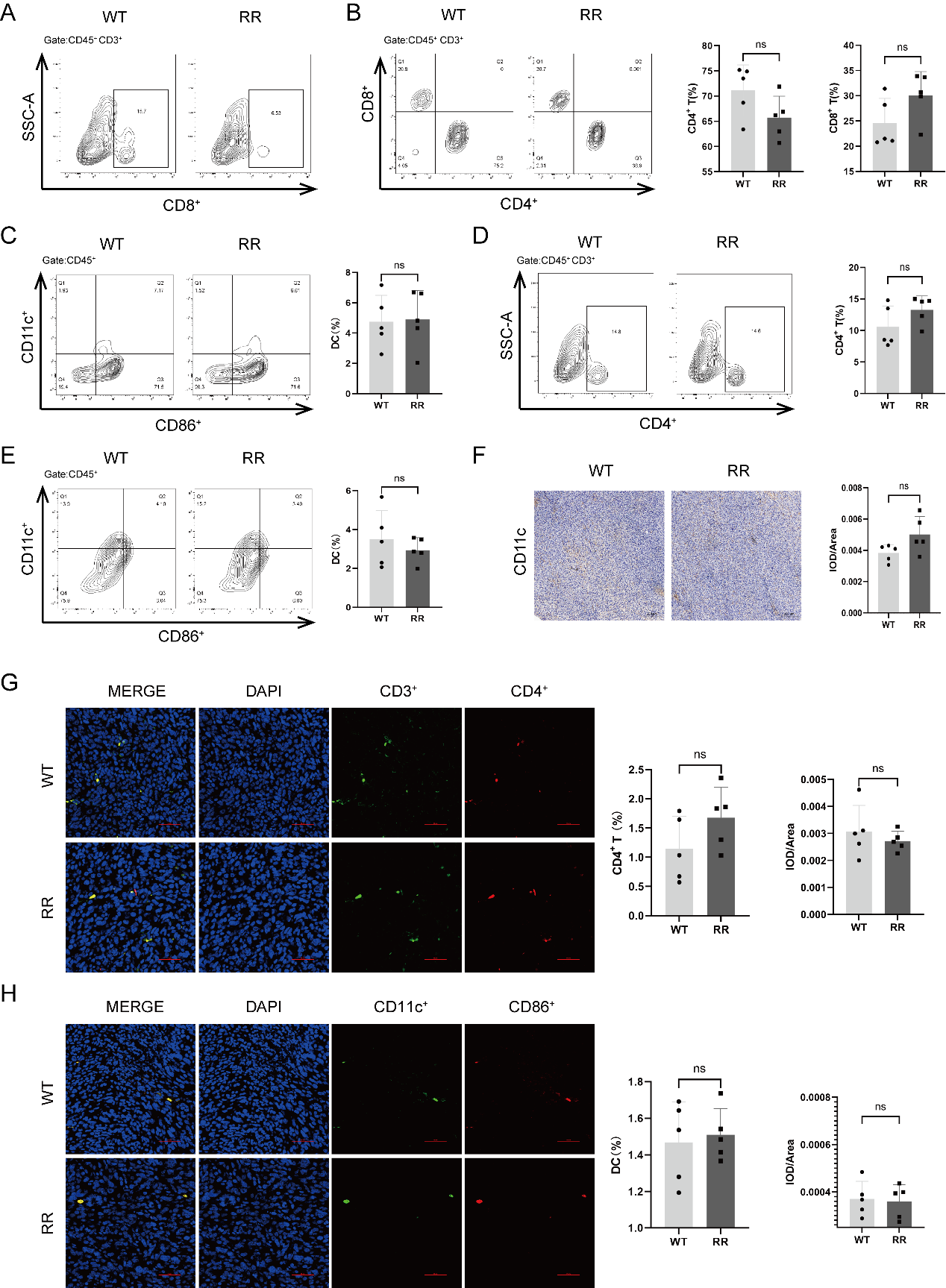


**Figure S3.** **(A).** Flow cytometry analysis of the proportion of CD8^+^ T cells in the tumor tissues. Proportion of CD4^+^ T cells, CD8^+^ T cells **(B)** and DCs **(C)** in the blood of mice analyzed by flow cytometry. Flow cytometry analysis of the proportion of CD4^+^ T cells **(D)** and DCs **(E)** in the tumor tissues from mice. **(F).** Immunohistochemical analysis of protein expression levels of CD11c in mouse tissues. **(G-H).** Immunofluorescence analysis of CD4^+^ T cells and DCs in mouse tumor tissues. Data indicate the mean ± SD. *p < 0.05, **p < 0.01, and ***p < 0.001, by 2-tailed Student’s t test or one-way ANOVA. N=5.


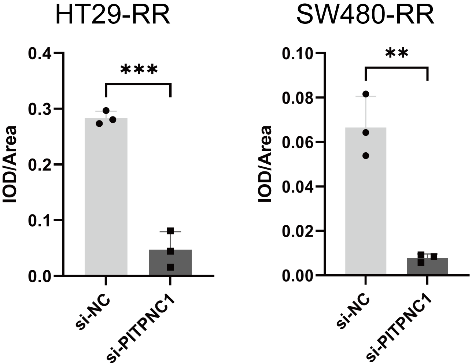


**Figure S4.** Immunofluorescence co-localization of PITPNC1 and FASN proteins in cells. Data indicate the mean ± SD. *p < 0.05, **p < 0.01, and ***p < 0.001, by 2-tailed Student’s t test or one-way ANOVA. N=3.


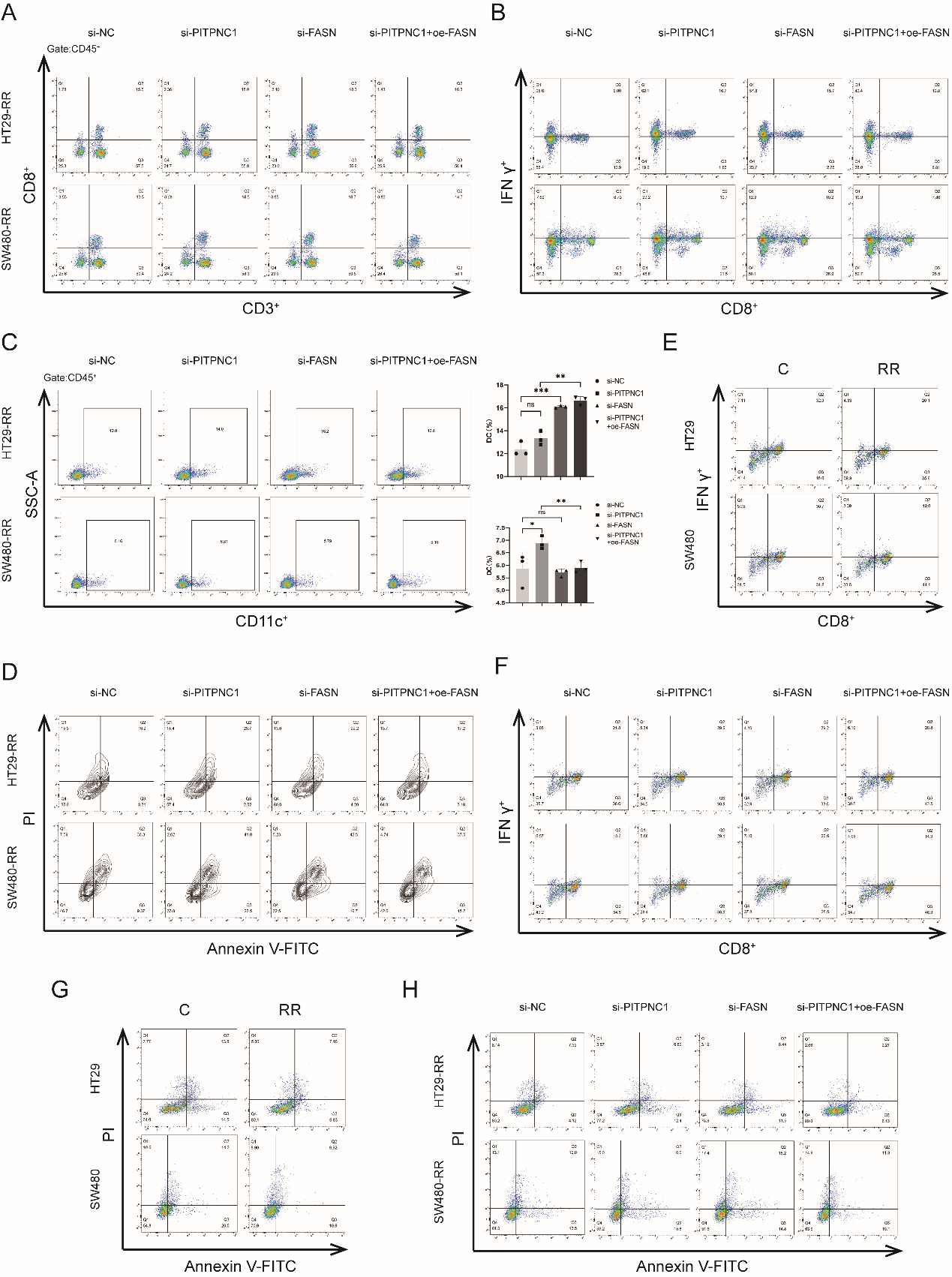


**Figure S5. (A).** The analysis of the proportion of CD8^+^ T cells after co-culturing PBMCs with tumor cells performed by flow cytometry. **(B).** After co-culturing PBMCs with tumor cells, the flow cytometry analysis demonstrated the changes in the proportion of IFN-γ^+^ CD8^+^ T cells. **(C).** Analysis of the alterations in the proportion of DCs exhibited by flow cytometry after co-culturing PBMCs with the tumor cells. **(D).** The flow cytometry analysis of the apoptosis rate of tumor cells in co-cultivation of PBMCs and tumor cells. **(E-F).** After co-culturing CD8^+^ T cells with tumor cells, the flow cytometry analysis demonstrated the changes in the proportion of IFN-γ^+^ CD8^+^ T cells. **(G-H).** The flow cytometry analysis of the apoptosis rate of tumor cells in co-cultivation of CD8^+^ T cells and tumor cells. Data indicate the mean ± SD. *p < 0.05, **p < 0.01, and ***p < 0.001, by 2-tailed Student’s t test or one-way ANOVA. N=3.


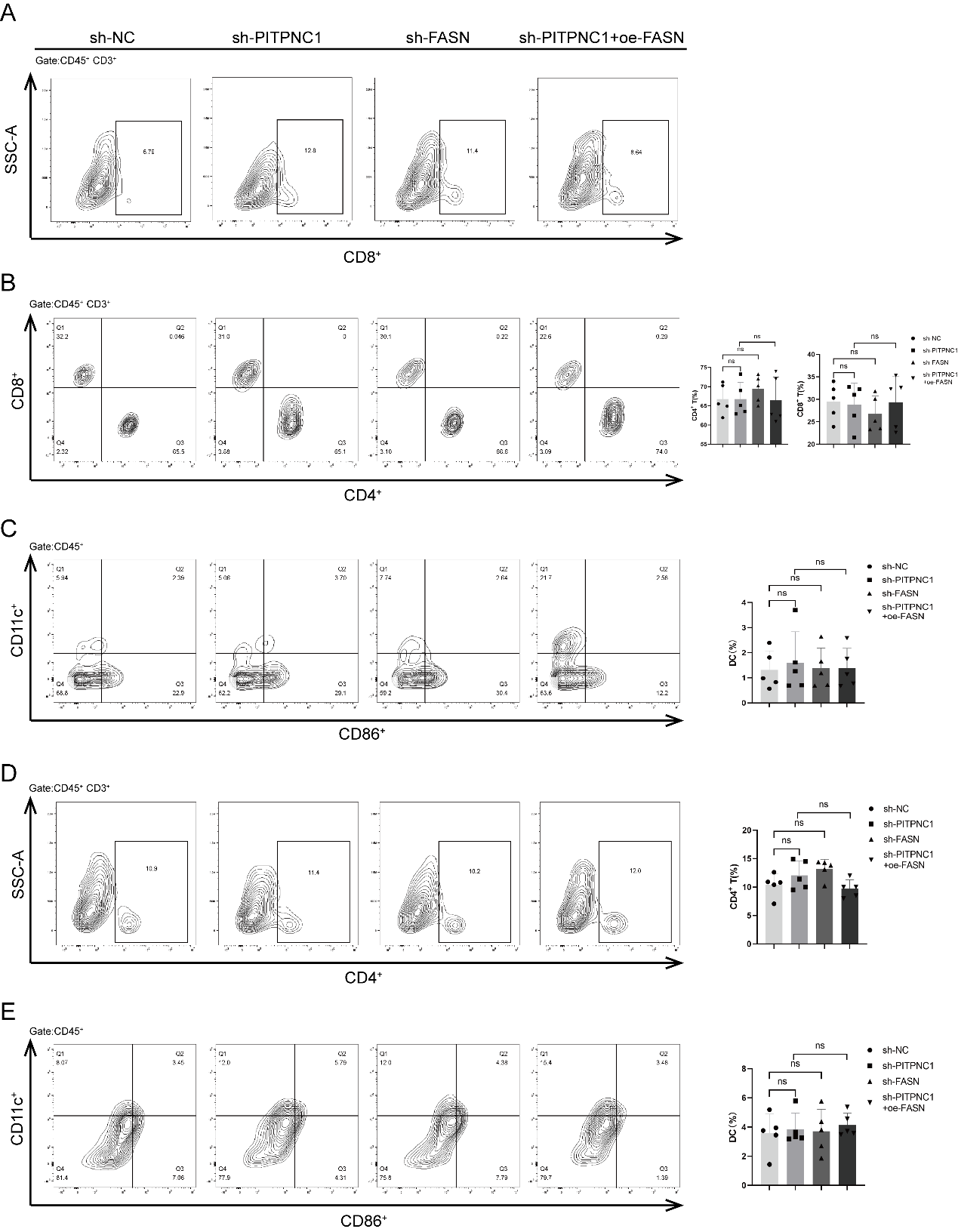


**Figure S6. (A).** Analysis of the proportion of CD8^+^ T cells in the tumor tissues using flow cytometry. Analysis of murine blood CD4^+^ T cells, CD8^+^ T cells **(B)** and DCs **(C)** proportions using flow cytometry. **(D-E)** Flow cytometry analysis of CD4^+^ T cells and DCs proportions in murine tumor tissues. Data indicate the mean ± SD. *p < 0.05, **p < 0.01, and ***p < 0.001, by 2-tailed Student’s t test or one-way ANOVA. N=5.


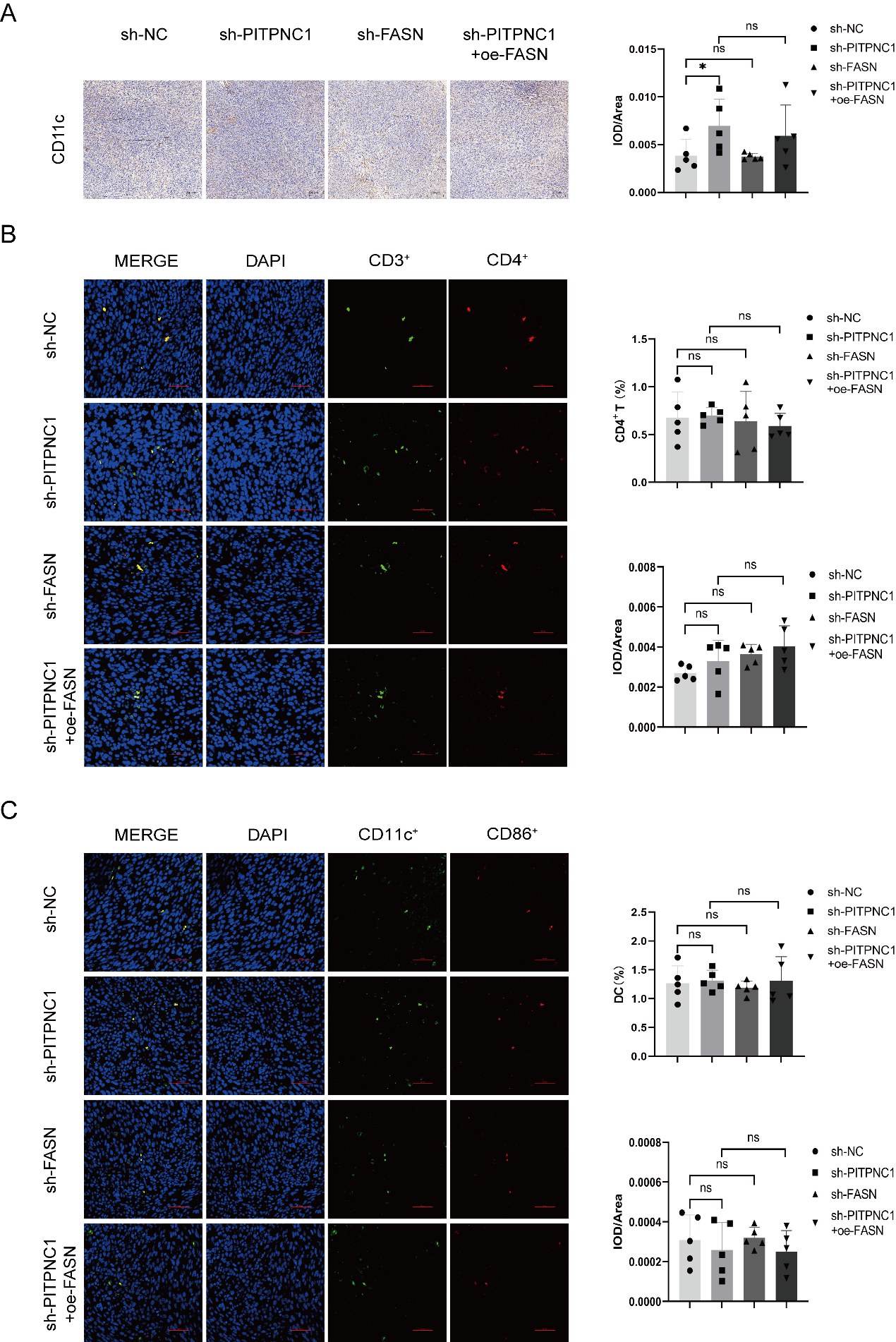


**Figure S7. (A).** Immunohistochemical analysis of protein expression levels of CD11c in mouse tumor tissues. **(B-C).** Immunofluorescence analysis of CD4^+^ T cells and DCs in mouse tumor tissues. Data indicate the mean ± SD. *p < 0.05, **p < 0.01, and ***p < 0.001, by 2-tailed Student’s t test or one-way ANOVA. N=5.
